# Sustainable Bacterial Cellulose Production Using Low-Cost Fruit Wastewater Feedstocks

**DOI:** 10.3390/nano15040271

**Published:** 2025-02-11

**Authors:** Cláudia Mouro, Arlindo Gomes, Ana P. Gomes, Isabel C. Gouveia

**Affiliations:** 1 Aeronautics and Astronautics Research Center, Faculty of Engineering, University of Beira Interior, 6200-001 Covilhã, Portugal; claudia.mouro@ubi.pt (C.M.); anapaula@ubi.pt (A.P.G.); 2 FibEnTech Research Unit, Faculty of Engineering, University of Beira Interior, 6200-001 Covilhã, Portugal; catu@ubi.pt

**Keywords:** bacterial cellulose, fermentation, low-cost feedstock, membrane fractionation, membrane separation technology, fruit processing wastewater

## Abstract

Bacterial cellulose (BC) is a versatile biopolymer prized for its remarkable water absorption, nanoscale fiber architecture, mechanical robustness, and biocompatibility, making it suitable for diverse applications. Despite its potential, the high cost of conventional fermentation media limits BC’s scalability and wider commercial use. This study investigates an economical solution by utilizing fractions from fruit processing wastewater, refined through sequential membrane fractionation, as a supplement to commercial HS medium for BC production. BC films were thoroughly characterized using Fourier transform infrared spectroscopy (FTIR), transmission electron microscopy (TEM), X-ray diffraction (XRD), differential scanning calorimetry (DSC), and assessments of mechanical properties and water holding capacity (WHC). FTIR confirmed the BC structure, while TEM validated its nanofibrillar 3D network. XRD analysis revealed a slight increasing trend in crystallinity with the addition of wastewater fractions, and DSC revealed a slight increase in thermal stability for F#6. Adding these fractions notably improved the BC films’ tensile strength, Young’s modulus, and WHC. Overall, the results underscore that fruit processing wastewater fractions can serve as a cost-efficient, eco-friendly alternative to traditional fermentation media. This approach supports circular economy principles by lowering reliance on intensive wastewater treatments, promoting waste valorization, and advancing sustainable production methods for high-value biopolymers.

## 1. Introduction

Cellulose is a polysaccharide composed of linear chains of β-1,4-glucan units, which form ribbon-like structures through hydrogen bonding. It is the most prevalent macromolecule and a key component of plant cell walls, providing essential mechanical support [1,2]. Despite this structural complexity, cellulose has garnered significant interest as a biopolymer due to rising environmental concerns about petroleum-based plastics and the growing demand for sustainable alternatives. Its biodegradability, renewability, and low environmental impact make it a promising candidate for application in biodegradable packaging, eco-friendly construction materials, and biomedical devices [1,2,3].

In addition to plant-derived cellulose, various aerobic acetic bacteria, including species from the genera *Komagataeibacter*, *Gluconacetobacter*, and *Acetobacter* can synthesize cellulose as an exopolysaccharide secreted during their metabolic processes. Among these, *Komagataeibacter xylinus* (*K. xylinus*) stands out due to its exceptional tolerance to acetic acid and its highly efficient production of bacterial cellulose (BC), which has been the focus of extensive research [1,4,5,6,7].

The rapid synthesis of cellulose by bacteria demonstrates significantly higher productivity compared to plant-derived cellulose [1]. This efficient production is closely linked to BC’s unique structural organization [1,6,8,9]. Unlike plant-derived cellulose, BC lacks pectin, lignin, and hemicellulose, which contributes to its exceptional properties, such as mechanical strength, crystallinity, water-holding capacity (WHC), degree of polymerization, chemical stability, and biological adaptability, making it advantageous for various applications, including food packaging, pharmaceuticals, medical industries, cosmetics, biotextiles, electrical and magnetic materials, and bioremediation in wastewater treatment [1,4,5,6,10,11,12].

BC’s biosynthesis occurs in the bacteria’s periplasmic space, where cellulose forms protofibrils that organize into ribbon-like microfibrils, essential for BC film formation [1,13]. Nevertheless, BC production benefits from the symbiotic interactions between bacteria and yeasts. In kombucha, a popular probiotic beverage used for laboratory-scale BC production, the symbiotic community of bacteria and yeasts (SCOBY—symbiotic culture of bacteria and yeast) involves cooperative and competitive interactions that benefit both types of microorganisms [1,4,14]. Yeasts produce the enzyme invertase, an enzyme that hydrolyzes sucrose into glucose and fructose, providing a carbon source for all microorganisms. Simultaneously, bacteria produce organic acids, such as gluconic and acetic acids, and form a gel-like cellulose film at the liquid–air interface, which acidifies the medium and creates a protective barrier. Additionally, ethanol produced by yeasts further stimulates cellulose biosynthesis, promoting BC film production [1,14].

Nonetheless, despite BC’s promising applications, its production remains expensive, mainly due to fermentation media and operational costs. However, recent advancements in cultivation strategies, fermentation processes, medium compositions, and bioreactor configurations, including rotating disc systems, are being developed to address these cost challenges and improve production efficiency. Nevertheless, traditional BC production still depends on commercial media, such as the Hestrin–Schramm (HS) medium, which requires expensive carbon (C) sources like glucose and nitrogen (N) sources such as yeast extract and peptone [4,5,14,15].

Therefore, selecting the most beneficial culture medium components is crucial for optimizing BC yield and production efficiency. While the HS medium typically uses glucose and peptone-yeast extract, alternative C sources like fructose, galactose, and mannose have also demonstrated potential for supporting microorganism growth. However, the high cost of these alternative C sources remains a substantial obstacle to widespread industrial and academic implementation of BC [1,4,14,15].

To address this issue, researchers have explored a range of industrial waste as alternatives to conventional media. These include agricultural by-products such as corn stalks [16], corncobs [17], and sugarcane bagasse [17], sugar beet and sugarcane molasses [18,19], dry olive mill residue [20], brewing by-products [21], lignocellulosic biomass wastes, rice husks [22], konjac powder [23], acerola waste [24], tobacco waste extract [25], cotton-based waste textiles [26], waste from fruit processing, and wastewater from candy processing [22].

In particular, the food processing industry, with its significant water consumption and substantial wastewater generation, presents a major environmental challenge [27,28]. The wastewater is characterized by high levels of readily biodegradable organic matter, typically quantified in terms of biological and chemical oxygen demand (BOD and COD), along with high concentrations of suspended solids, N, and phosphorus (P) [29,30]. Mitigating the environmental impact of these wastewaters requires complex and costly physical, chemical, and biological treatment processes [31]. Moreover, the management and disposal of such wastewater impose significant financial and economic burdens [32]. Therefore, these conventional treatment methods are unsustainable, consuming substantial resources and overlooking opportunities for recovering valuable energy sources (e.g., methane, biofuels, and biohydrogen) and producing high value bioproducts (e.g., organic acids, alcohols, proteins, microbial biomass, and biopolymers) [33,34,35]. In addition, they do not align with circular economy principles, emphasizing resource efficiency and waste valorization [29].

Thus, this research study assesses the feasibility of using fruit processing wastewater—by-products obtained during fruit processing like fruit juice processing, handling fruit flesh, washing, peeling, and other steps involved in fruit-based product manufacturing [36,37]—for BC production, using a microbial consortium derived from kombucha.

To achieve this, a low-cost and efficient approach based on sequential membrane fractionation with increasing selectivity was employed to effectively separate and concentrate essential nutrients for BC production from raw wastewater.

Membrane technologies are widely used in industry to treat wastewater before disposal due to potential environmental concerns. In this context, adopting these technologies not only addresses environmental requirements but also offers an opportunity for waste valorization. For example, in the food processing industry, membrane fractionation is applied for concentration, clarification, and purification of food products [38,39]. Additionally, membrane technologies provide significant environmental and economic benefits by enabling the efficient recovery and reuse of by-products and water, thereby helping industries like pharmaceuticals, biotechnology, chemicals, and textiles comply with discharge regulations [40,41,42]. Nevertheless, the effectiveness of this method depends on membrane technologies, ranging from microfiltration (MF) to reverse osmosis (RO), which are selected based on molecular size, charge, or chemical affinity [43].

Considering these advantages, the present research work revealed that applying fruit processing wastewater from mixtures of fruit and cereals used in yogurt production for BC synthesis is a promising approach to addressing environmental and economic challenges. Sequential membrane fractionation demonstrated that the resulting fractions, particularly F#6, instead of being discarded, can be effectively recycled as a source of essential nutrients for BC production, contributing to reducing dependence on commercial culture media. Moreover, this approach aligns with circular economy principles by enabling the efficient reuse of residues, transforming waste disposal into a resource recovery opportunity, and promoting more sustainable and cost-effective biopolymer production.

## 2. Materials and Methods

### 2.1. Materials

Kombucha Original Bio was acquired from Freshness Diagonal, Lda (Montijo, Portugal). Glucose, peptone, yeast extract, disodium hydrogen phosphate (Na_2_HPO_4_), sodium hydroxide (NaOH), uranyl acetate, and sodium metabisulfite were provided by Sigma-Aldrich (St. Louis, MO, USA). Citric acid and acetic acid were purchased from Panreac (Barcelona, Spain). 3,5-dinitrosalicylic acid (DNS) and potassium sodium tartrate tetrahydrate were purchased from Fluka (Sintra, Portugal).

### 2.2. Membrane Fractionation Technique from Fruit Processing Wastewater

The fruit processing raw wastewater, kindly provided by Frulact S.A. (Maia, Portugal), a fruit processing industry specialized in the development and processing of fruit-based preparations for application in the food industry, was collected in January 2022 and subjected to sequential membrane fractionation to attend as an alternative feedstock for BC production. The raw wastewater from fruit and cereal mixtures used in yogurt production, containing suspended solids and soluble compounds, was stored at 5 ± 1 °C until being used.

**Figure 1:**
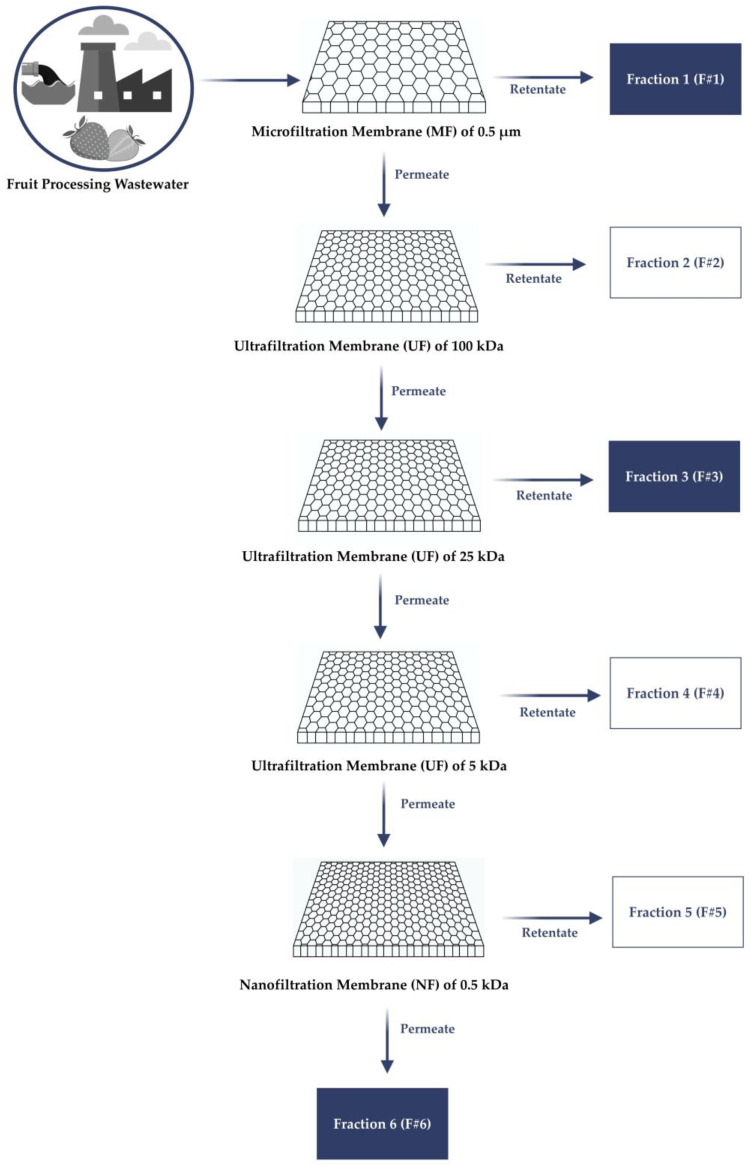
Schematic representation of the sequential membrane fractionation process. Fraction 1 (F#1): retentate from microfiltration (MF) containing suspended solids; Fraction 2 (F#2): retentate from 0.50 µm–100 kDa fractionation; Fraction 3 (F#3): retentate from 25–100 kDa fractionation; Fraction 4 (F#4): retentate from 5–25 kDa fractionation; Fraction 5 (F#5): retentate from 0.5–5 kDa fractionation; Fraction 6 (F#6): permeate from <0.5 kDa fractionation.

A combination of MF, ultrafiltration (UF), and nanofiltration (NF) membrane modules, produced by Alfa Laval (Nakskov, Denmark) with a spiral wound configuration and a membrane area of 0.55 m^2^ each, was used for sequential membrane fractionation (please see the illustrative representation of the sequential membrane fractionation process in Figure 1). Firstly, the raw wastewater was subjected to MF using an appropriate membrane with a pore size of 0.5 µm (MFP5) to separate suspended solids from soluble fractions. The resultant retentate from MF (F#1) was collected for BC production testing, while the permeate-containing soluble compounds proceeded to the subsequent fractionation phase. Concerning that, the permeate from MF was subjected to sequential UF using membranes with pore sizes of 100 kDa (GR40PP) and 25 kDa (GR60PP) aimed at concentrating and purifying specific nutrients required for BC production. The resulting retentate from the 25 kDa UF membrane (F#3) was used in this research study as a substrate to supplement HS medium for CB production. Then, the 25 kDa UF permeate was further processed through 5 kDa UF membranes (GR90PP) and 0.5 kDa NF membrane (NFT). The NF membrane, which exhibits over 99% rejection of 2 g/L MgSO_4_ aqueous solution, was used to improve the separation. The permeate obtained from the 0.5 kDa NF membrane (F#6) was also collected as an alternative feedstock for testing BC production.

Regarding the operational conditions, the membrane modules were sequentially installed and operated in a Labstak M20 pilot system from DSS (Silkeborg, Denmark), following the method described by Gomes et al. (2005) [44]. Operational conditions included a constant temperature of 20 ± 2 °C, maximum tangential flow velocity to minimize concentration polarization, and pressures of 1 bar for MF, 1 to 5 bar for UF, and 10 bar for NF.

Before each use, the membrane modules were pressurized at the maximum recommended operating pressure for 3 h using pure water to clean and compact the membranes, which further contributed to the prevention of fouling and scaling. Hydraulic permeability to pure water (L h^−1^ m^−2^ bar^−1^) was then measured. The hydraulic permeability correlated linearly with pressure, and these values were used as references for subsequent cleaning and recovery processes. 

Moreover, before each fractionation stage, steady-state operational conditions were achieved after 15–30 min, after which permeate collection began. The volumetric reduction factor (VRF), representing the ratio of feed volume to retentate volume, was set to 1.5 (e.g., 100 L of feed solution processed to obtain 67 L of permeate).

Table 1 summarizes the membranes’ specifications, including permeability values measured after the stabilization and compaction of the membrane structure.

### 2.3. Determination of the Carbon (C) and Nitrogen (N) Content in the Fruit Processing Wastewater

The suitability of fruit processing wastewater as a supplement or replacement for the HS medium in BC production was evaluated from the COD (indicative of the amount of organic matter), the DNS colorimetric method (indicative of the reducing sugar content, which can be used as a C source for microbial growth), and the total dissolved N content in the raw wastewater and the selected fractions. Briefly, the COD of raw wastewater was quantified using closed reflux and titrimetric methods [45], while total dissolved N was determined with cuvette tests LCK238 (5–40 mg N L^−1^) from Hach-Lange (Düsseldorf, Germany) after filtration through 0.45 μm membrane filters. Additionally, COD and dissolved N in the selected fractions were quantified by mass balance analysis after the suspended solids were removed through the MF membrane. Given the high suspended solids content in the raw wastewater, this approach was chosen to ensure a more reliable measurement. In turn, the reducing sugar content was measured using the DNS assay, following the protocol by Miller (1959) [46,47]. The concentration of reducing sugars in the selected fractions was then determined using a standard glucose absorbance curve at 540 nm (y = 1.2357x − 0.751, R^2^ = 0.9942) over a concentration range of 0–20 g/L. For each measurement, 2 mL of the sugar solution was mixed with 2 mL of the DNS reagent in a test tube. The tubes were vortexed, boiled in a water bath for 10 min, rapidly cooled to room temperature in an ice bath, and the absorbance was measured at 540 nm using a UV-Vis spectrophotometer.

### 2.4. Inoculum Preparation for BC Production

The starter culture, or SCOBY, was sourced from a commercial Kombucha Original Bio beverage containing various bacterial and yeast species. To prepare the inoculum, 10% (*v*/*v*) of the kombucha culture medium was added to HS broth, which consisted of 2.0% (*w*/*v*) glucose, 0.5% (*w*/*v*) peptone, 0.5% (*w*/*v*) yeast extract, 0.27% (*w*/*v*) Na_2_HPO_4_, and 0.15% (*w*/*v*) citric acid. The pH of the medium was adjusted to 6.0, and the mixture was incubated at 30 °C for 120 h to allow sufficient bacterial cell growth. After the incubation period, the inoculum was vigorously agitated to release the cells that were embedded in the gel-like BC biofilm formed at the air–liquid interface. The resulting cell suspension was then used to inoculate the different flasks.

### 2.5. Preparation of Fermentation Medium and BC Production Using the Fruit Processing Wastewater Fractions

In this research study, various fermentation media were prepared using different formulations. The conventional HS fermentation medium was used as the standard to produce control BC samples. Otherwise, the fermentation media were supplemented with the selected fractions F#1, F#3, and F#6, obtained from raw wastewater after sequential membrane fractionation, to replace or supplement the C and N sources in the commercial medium. In particular, the HS medium was supplemented with 5.0% (*v*/*v*) F#1, 10.0% (*v*/*v*) F#1, 5.0% (*v*/*v*) F#3, 10.0% (*v*/*v*) F#3, 5.0% (*v*/*v*) F#6, and 10.0% (*v*/*v*) F#6, respectively. Moreover, 10.0% (*v*/*v*) of the previously prepared inoculum was added to each 250 mL Erlenmeyer flask, with a final volume of 200 mL to initiate BC biosynthesis. Prior to fermentation, the final pH of each fermentation media, including those supplemented with fruit wastewater fractions, was adjusted to 6 by adding 1 N acetic acid, and the flasks were then incubated at 30 °C for 7 days under static conditions. Table 2 summarizes the composition of each formulation, including the designation of the medium.

### 2.6. Harvesting and Purification of BC Films

At the end of the incubation period, the gel-like BC films that formed at the liquid–air interface of the medium were collected and purified. This purification process included washing the BC films with 0.1 M NaOH at 80 °C for 1 h to eliminate cellular debris and medium components. Subsequently, the films were rinsed with distilled water until achieving a neutral pH of 7, and the purified BC was dried to a constant weight at 60 °C.

### 2.7. Determination of BC Dry Weights

The BC’s dry weight was determined after it was washed with distilled water and dried at 60 °C until a constant weight was achieved, using an analytical balance (Mettler Toledo, Switzerland) with a readability of 0.01 mg.

### 2.8. Productivity Parameters of BC Films Using Fruit Processing Wastewater Fractions

The production yield of BC films is a critical parameter for evaluating fermentation performance. In this study, BC production yield in the HS commercial medium and HS media supplemented with the selected fractions was determined by calculating the ratio of BC dry weight to the initial weights of the C and N sources in each fermentation medium. The yield based on the C source was calculated to reflect the amount of BC synthesized from the C source, while the yield based on the N source was considered to represent the indirect relationship between N availability and BC synthesis. Additionally, BC productivity per volume of fermentation medium was also estimated to assess the efficiency of the process. The production yield of BC was determined using Equations (1)–(3):

(1) $$Yield\;by\;carbon\;source\;\left(\%\right)=\frac{W_{dry}}{C}\times 100$$

(2) $$Yield\;by\;nitrogen\;source\;\left(\%\right)=\frac{W_{dry}}{N}\times 100$$

(3) $$BC\;Yield\;\left(g/L\right)=\frac{W_{dry}}{V}$$

where *W_dry_* is the BC dry weight, *C* and *N* represent the weights of the *C* (i.e., glucose and reducing sugars present in the wastewater fractions) and *N* sources (i.e., yeast extract, peptone, and total dissolved nitrogen in the wastewater fractions), respectively, and *V* is the volume of the medium used in BC production.

Furthermore, for comparative analysis, the BC production rate was estimated using the BC yield (*g/L*) and the fermentation period in days (*D*), as described by Equation (4):

(4) $$BC\;production\;rate\;\left(g/L/day\right)=\frac{BC\;Yield}{D}$$

### 2.9. Analysis of the BC Properties

The morphological, physicochemical, and mechanical properties of dry BC films were analyzed.

#### 2.9.1. Thickness and Opacity Measurements of the BC Films

The thickness of the BC films was measured using a micrometer (Adamel Lhomargy MI20, Roissy-en-Brie, France). Each BC sample was measured ten times, and the thickness was determined as the mean of these measurements. The opacity of BC films was assessed using a UV–Vis spectrophotometer (UV-300 Unicam), following a previously established method [48,49]. BC dry samples were prepared to fit perpendicularly into a glass cuvette, while an empty glass cuvette was used as the reference. The opacity of BC films was calculated using the following Equation (5):

(5) $$Opacity=\frac{{Abs}_{600}}{T}$$

where ${Abs}_{600}$ represents the absorbance of dry BC films at 600 nm and T represents the thickness of the BC samples in millimeters (mm).

#### 2.9.2. Visual Appearance of BC Films

The appearance of the BC films was evaluated using CIELAB color coordinates, and their whiteness index (*WI*) was calculated. The L*, a*, and b* values were recorded with a Datacolor 110 spectrophotometer (Datacolor Company, Lawrenceville, NJ, USA) under illuminant D65 and a 10° standard observer. These values were then used to calculate the *WI*, as shown in Equation (6):

(6) $$WI=100-[{\left(100-L^{*}\right)}^{2}+{\left(a^{*2}+b^{*2}\right)]}^{1/2}$$

L* indicates the lightness of a color, a* represents the green to red spectrum (–a* *=* greener, +a* *=* redder), and b* corresponds to the blue to yellow color (–b* *=* bluer, +b* *=* yellower). Measurements were taken at a minimum of three random points per sample and averaged. Additionally, the color change (Δ*E*) was calculated using Equation (7):

(7) $$\Delta E=\sqrt{{\left(L^{*}-L_{0}\right)}^{2}+{\left(a^{*}-a_{0}\right)}^{2}+{\left(b^{*}-b_{0}\right)}^{2}}$$

where $L_{0}$, $a_{0}$, and $b_{0}$ are the coordinates corresponding to the control (HS medium), against which the BC films were compared to determine the color change resulting from each fermentation medium supplemented with the fractions obtained from fruit processing raw wastewater after sequential membrane fractionation.

#### 2.9.3. Fourier Transform Infrared Spectroscopy (FTIR)

The BC films’ FTIR spectra were obtained at room temperature using a Thermo-Nicolet iS10 FT-IR spectrometer equipped with an attenuated total reflectance (ATR) diamond module to analyze their functional groups. Each sample was scanned over a wavenumber range from 4000 to 550 cm^−1^ with a resolution of 4 cm^−1^, accumulating 64 scans per sample.

#### 2.9.4. Transmission Electron Microscopy (TEM)

The morphology of the BC nanofibrils were evaluated using Transmission Electron Microscopy (TEM; HITACHI HT7700). For this purpose, BC suspensions were stained with 2.0% (*w*/*v*) uranyl acetate for 5 min, placed on nickel grids with formvar carbon, and dried at room temperature. TEM images were then acquired at an accelerating voltage of 80 kV and at a magnification of 3000×. The diameters of the BC nanofibrils were measured using ImageJ software (ImageJ 2.0.0 NIH Image, USA), *n* = 50.

#### 2.9.5. X-Ray Diffractometry (XRD)

The crystallinity of the BC films was determined by X-ray diffractometry (XRD) with a Rigaku DMAX III/C X-ray diffractometer equipped with a copper X-ray source (1.54 Å) operated at filament emission of 30 mA and voltage of 40 kV. The BC films were scanned in the 2θ range from 5° to 60° at a scanning rate of 1.2°/min. Background subtraction and smoothing were not applied to the diffraction patterns. The crystallinity index (*CrI*) of the produced BC was determined using the Segal method [50] with the following Equation (8):

(8) $$CrI\;\left(\%\right)=[(I_{110}-I_{am})/I_{110}\times 100$$

where $I_{110}$ represents the maximum diffraction intensity value of the lattice peak at 2θ found between 22° and 23°, and $I_{am}$ is the minimum diffraction intensity of the amorphous peak around 18° (between the (010) and (110) peaks) [50].

#### 2.9.6. Differential Scanning Calorimetry (DSC) Measurements

DSC thermograms of the BC films were obtained using a differential scanning calorimeter (DSC 204 Phoenix, Netzsch, Germany) over 30 to 400 °C, with a heating rate of 5 °C/min. Approximately 5 mg of each BC sample was placed into aluminum pans and sealed with airtight lids. An empty aluminum pan served as the reference.

#### 2.9.7. Mechanical Properties

Uniaxial tensile tests were conducted on BC films using a Universal tensile test machine (Adamel Lhomargy Division d’Instruments Model DY-35, France) equipped with a 100 N load cell. Rectangular BC samples measuring 30 mm in length and 10 mm in width were prepared. The gauge length between the clamps was set to 15 mm, and the tests were performed at a testing speed of 1 mm/min at room temperature. Five specimens of each film were tested to evaluate maximum tensile strength (MPa), Young’s modulus (MPa), and deformation at break (%).

#### 2.9.8. Water Holding Capacity (WHC)

The WHC of the BC films was determined using the sieve shake method, following procedures outlined by Ul-Islam et al. (2012) and Barshan et al. (2019) [51,52]. Initially, dried BC samples were immersed in distilled water until fully rehydrated. They were then placed in a sieve, subjected to two quick shakes to remove surface water, and subsequently weighed. Afterward, the BC films were dried until a constant weight was achieved. WHC was calculated using the Equation (9):

(9) $$WHC\;\left(\%\right)=\frac{W_{Wet}-W_{Dry}}{W_{Wet}}$$

where $W_{Wet}$ is the weight of the BC samples after shaking off excess water, and $W_{Dry}$ is the weight of the BC samples after completely drying.

### 2.10. Statistical Analysis

All experiments were performed three times unless stated otherwise. Results are presented as the mean ± standard deviation (SD) of three experiments. Statistical significance was determined using one-way or two-way ANOVA, followed by Tukey’s multiple comparisons test (*p* < 0.05), analyzed with GraphPad Prism 6 software.

## 3. Results and Discussion

### 3.1. Determination of the Carbon (C) and Nitrogen (N) Content in the Fruit Processing Wastewater

Various studies have previously reported the use of membrane sequences with increasing selectivity. For instance, domestic wastewater has been used to establish a correlation between particle size distribution and COD as an index for biological treatability [53]. Additionally, this methodology has also been employed to assess the toxicity of olive mill wastewater fractions [54] and investigate the susceptibility to oxidation of molecular size fractions of refractory organic compounds from aged raw landfill leachate [55]. These examples underscore the importance of understanding the organic load characteristics to optimize waste treatment processes and explore effective waste valorization strategies.

Concerning that, in this research study, the COD of the fruit processing raw wastewater and the selected fractions obtained through sequential membrane fractionation with increasing selectivity were evaluated to estimate the organic matter content and the efficiency of the fractionation process.

The results showed a high COD value of 23.32 ± 1.64 g/L for the retentate of the MF containing the suspended solids (F#1), indicating a substantial organic load typical of fruit processing wastewater [56]. However, the retentate from the 25 kDa UF membrane (F#3) and the permeate obtained from the 0.5 kDa NF membrane (F#6) obtained COD values of 15.26 ± 0.51 g/L and 0.76 ± 0.03 g/L, respectively. These findings suggest that membrane fractionation effectively reduced the organic load in the wastewater, revealing the efficacy of the process in producing a permeate of significantly lower organic content suitable for further reuse in various applications.

In addition to measuring COD, reducing sugars were quantified in each selected fraction using the DNS assay to evaluate their potential as an alternative C source for replacing or supplementing the commercial HS medium used in BC production. Total dissolved N was also measured, as N sources are essential for microbial growth and cell development (Table 3 and Table 4).

Overall, the raw wastewater presented a high concentration of suspended solids (2.52 ± 0.09 g/L) and a higher pH of 11.5 ± 0.8, which may hinder BC film formation, as seen in Table 3. Previous studies have shown that the optimal pH range for BC production is between 4 and 7 [22]. Additionally, the selected fractions from raw wastewater after sequential fractionation through increasingly selective membranes were analyzed, as seen in Table 4. F#1 retentate also comprised a high concentration of suspended solids (3.98 g/L), which strongly restrained the reliability of the determinations of dissolved N content in this fraction. Moreover, the high concentration of suspended solids and the complex composition of F#1, which exhibited a high COD of 23.32 ± 1.64 g/L, may impede the formation of the BC film by interfering with microbial activity and disrupting the uniformity of film development.

On the other hand, for F#3, the COD was 15.26 ± 0.51 g/L, indicating a significant reduction in organic load compared to F#1. The reducing sugars content was 0.85 ± 0.11 g/L, similar to F#1 (0.78 ± 0.09 g/L), suggesting that the membrane fractionation process did not significantly affect the concentration of these sugars. However, the reduction in COD indicates that F#3 contains fewer complex organic compounds, which could favor BC’s growth by reducing potential microbial activity inhibitors. Moreover, the total dissolved N content in F#3 was 6.18 ± 0.15 mg/L, comparable to the raw wastewater (6.44 ± 0.31 mg/mL). Therefore, the lower COD may create more favorable conditions for forming the BC film than F#1 despite the similar values of reducing sugars.

Particularly, the COD value was significantly lower for F#6 (0.76 ± 0.03 g/L), indicating that the NF membrane effectively removed most of the organic matter. Notably, the measured reducing sugars in this fraction were 0.74 ± 0.06 g/L, suggesting that a significant amount of the remaining organic matter in F#6 consists of reducing sugars, which may primarily include smaller compounds such as glucose that are less efficiently removed by the NF process. These smaller sugars are more easily metabolized by bacteria during BC production, potentially enhancing microbial activity and influencing the overall efficiency of BC production. In addition, the N content (2.70 ± 0.07 mg/L) was also reduced compared with the raw wastewater and the F#3, suggesting that the NF process effectively reduced the N content, likely removing both organic and some inorganic nitrogenous compounds, as seen in Table 4.

In addition, the pH of the selected fractions, ranging between 5.98 ± 0.16 and 6.70 ± 0.23 (please see Table 4), were within the optimal range for BC production, highlighting its importance as a factor affecting both the BC mechanical properties and production efficiency [6,22].

Further, when compared to the commercial HS medium, the reducing sugars of the selected fractions (F#1: 0.78 ± 0.60 g/L; F#3: 0.85 ± 0.11 g/L; and F#6: 0.74 ± 0.06 g/L) is lower than the C content of the HS medium, which contains 20 g/L of glucose. Additionally, the total dissolved N concentrations in the F#3 and F#6 fractions were also lower than the levels provided by the HS medium, which uses 0.5% (*w*/*v*) peptone and 0.5% (*w*/*v*) yeast extract. Nevertheless, sequential membrane fractionation shows promise in providing wastewater fractions with nutrients that could replace or supplement the HS medium, offering opportunities for cost reduction and waste valorization. However, adjustments to the nutrient composition may be necessary to optimize BC production efficiency.

### 3.2. Bacterial Cellulose (BC) Productivity Parameters Using Fruit Processing Wastewater Fractions

A microbial consortium of bacteria and yeast (SCOBY) derived from kombucha was used in this study to produce BC films. A proper balance between C and N sources is essential for supporting the metabolic activity and growth of the bacterial and yeast cells in SCOBY, although BC is predominantly synthesized from C sources [1].

Concerning that, commercial synthetic media for BC production often use high concentrations of C sources to optimize cell growth and metabolism, with glucose being the only C source in the HS commercial medium at a concentration of 2.0% (*w*/*v*). Although this high concentration is crucial for efficient BC production, it arouses significant costs. Thus, alternative C sources from industrial waste have been investigated to mitigate these costs [22]. In this study, fruit processing wastewater emerged as a promising solution due to its content of reducing sugars, which are essential for BC production, and its significant N content, crucial for synthesizing proteins, enzymes, and other cellular components that support microbial metabolism and cellulose production. Thus, the HS synthetic medium used for BC production, which includes yeast extract (0.5% (*w*/*v*)) and peptone (0.5% (*w*/*v*)) to provide essential N for microorganism growth and cell development, underscores the potential of wastewater as a viable alternative for supplying both nutrients.

**Figure 2:**
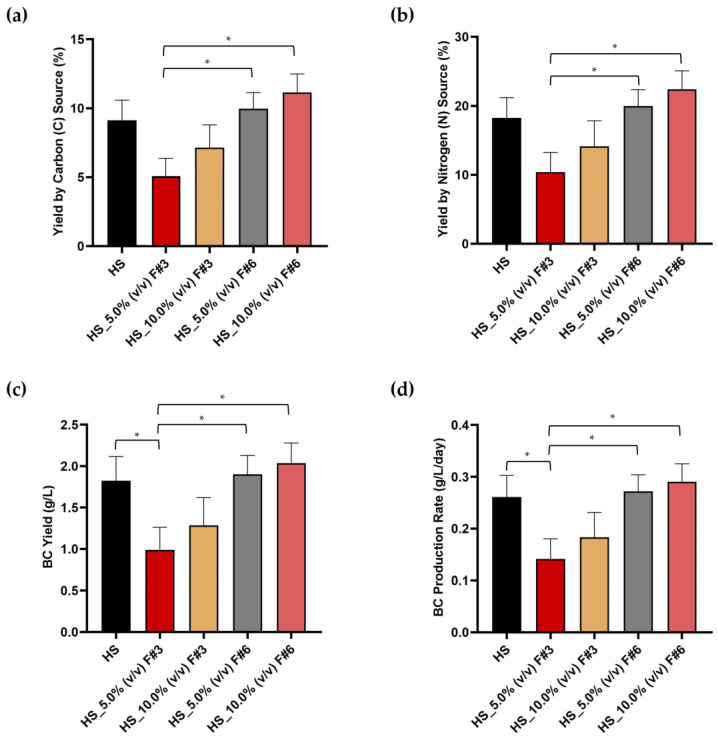
Yields of the bacterial cellulose (BC) films produced from Hestrin–Schramm (HS) medium supplemented with various fractions from fruit processing raw wastewater after membrane fractionation: (**a**) yield by carbon (C) source weight (%); (**b**) yield by nitrogen (N) source weight (%); (**c**) BC yield per volume of fermentation (g/L); and (**d**) BC production rate (g/L/day). (Data are presented as the mean ± standard deviation (SD), * *p* < 0.05).

The BC films produced in the HS medium (control) and HS media supplemented with 5.0% (*v*/*v*) and 10.0% (*v*/*v*) of the selected wastewater fractions after sequential membrane fractionation were collected after 7 days of static cultivation, washed, and dried to determine the BC production yields. However, the HS media supplemented with F#1, which primarily contains the suspended solids removed from the raw wastewater by the MF membrane, did not yield any BC films. This could be related to the fact that although essential nutrients may be present in F#1, they are mixed with a high amount of other non-specific components, including a high COD of 23.32 ± 1.64 g/L, which can interfere with the microbial processes needed for effective BC production [13]. Therefore, only the BC production yields in media supplemented with F#3 (the retentate of the intermediary size fraction of 25–100 kDa) and F#6 (the permeate of the NF) were compared to the yields in the commercial HS medium. The results were summarized in Figure 2.

The BC film produced from the commercial HS medium, which exclusively uses glucose as a C source and peptone and yeast extract as N sources, revealed a C yield of 9.126 ± 1.465% and a yield by N of 18.252 ± 2.931%, respectively, as seen in Figure 2a,b. These high yield values were expected, given the synthetic medium’s optimized environment for microbial growth and BC production and the high purity of the chemically graded C and N sources present in the HS medium. In turn, the BC films produced from HS medium supplemented with 5.0% (*v*/*v*) and 10.0% (*v*/*v*) F#3 exhibited carbon yields of 5.185 ± 1.430% and 7.028 ± 1.849% and nitrogen yields of 10.393 ± 2.865% and 14.115 ± 3.714%, respectively, as seen in Figure 2a,b. These yields were lower than those of the HS control, likely due to non-specific components in F#3 that may interfere with the microbial processes necessary for effective BC production. Although the COD of F#3 (15.26 ± 0.51 g/L) was lower than that of F#1 (23.32 ± 1.64 g/L), contaminants may still affect BC yields compared to the HS commercial medium. Furthermore, the HS medium supplemented with F#3 contained 0.85 ± 0.11 g/L of reducing sugars and 6.18 ± 0.15 mg/L of N, suggesting that the reduced BC yields are likely attributable to impurities in F#3, which diminish the effectiveness of these components compared to those in the HS medium. However, the BC films produced from the HS medium supplemented with 5.0% (*v*/*v*) and 10.0% (*v*/*v*) F#6 exhibited C and N yields comparable to or slightly better than those of the HS control, suggesting that the NF permeate could effectively supplement the commercial HS medium for BC production, as seen in Figure 2a,b. Although F#6 contains only 0.74 ± 0.06 g/L of reducing sugars and 2.70 ± 0.07 g/L of N, its effectiveness may be attributed to a balanced nutrient profile and a low COD of 0.76 ± 0.03 g/L, which indicates a significantly reduced level of non-specific components that could interfere with microbial activity. This implies that the quality of the nutrients in F#6, rather than their absolute concentrations, plays a crucial role in its potential as a viable alternative to supplement the HS medium for BC production.

A similar pattern was observed regarding BC productivity per fermentation volume, with the BC films produced from HS medium supplemented with F#6 showing higher yields than the HS control. Notably, the BC produced from HS medium supplemented with 10% (*v*/*v*) F#6 achieved a BC yield of 2.037 ± 0.243 g/L, surpassing the control HS, as seen in Figure 2c. This suggests that F#6 could be a promising supplement to the commercial HS medium, offering a favorable environment for BC production. In terms of BC production rate, F#6 also demonstrated superior performance. The production rate was highest with 10% (*v*/*v*) F#6, reaching 0.291 ± 0.035 g/L/day, compared to the HS control rate of 0.261 ± 0.042 g/L/day, as seen in Figure 2d. This indicates that F#6 not only enhances BC yield but also improves the production rate, suggesting that it could provide an efficient and cost-effective alternative to supplement the commercial HS medium, without compromising yield.

In turn, when the HS medium was supplemented with 5.0% (*v*/*v*) of F#6, yields were very similar to those obtained with the commercial HS medium, as seen in Figure 2. This suggests that even at lower concentrations, F#6 can effectively supplement the commercial medium, maintaining high productivity and offering versatility in medium formulation. Therefore, using F#6 from raw fruit processing wastewater reduces dependence on expensive commercial fermentation media and promotes the reuse of undervalued waste. This aligns with sustainability and circular economy principles, offering a cost-effective and eco-friendly alternative to conventional mediums while maintaining BC productivity.

Similarly, Srikandace et al. (2022) used rice-washed water (RW) and tofu processing wastewater (TW) as an alternative growth media to replace the traditional HS medium for BC production by *K. xylinus* [57]. The BC yield, calculated based on dry weight after 5 days of static fermentation at room temperature, was lower, with values of 0.50 g/L for RW, 0.73 g/L for TW, and 0.51 g/L for HS. However, after 10 days, the yields increased to 2.40 g/L for RW, 3.30 g/L for TW, and 2.22 g/L for HS [57]. In the present study, comparable yields to those reported for 10-day fermentations in HS and RW were achieved in just 7 days, particularly for BC films produced in HS medium (1.825 ± 0.293 g/L), HS medium supplemented with 5.0% (*v*/*v*) of F#6 (1.903 ± 0.368 g/L), and HS medium supplemented with 10.0% (*v*/*v*) of F#6 (2.037 ± 0.243 g/L). These findings suggest that our approach allows for a more efficient BC production process, achieving comparable yields in a shorter fermentation period.

### 3.3. Analysis of the BC Properties

#### 3.3.1. Visual Appearance of BC Films Produced from Fruit Processing Wastewater Fractions

The visual properties of BC films, encompassing opacity, whiteness, and color stability, were analyzed to assess their optical characteristics when produced using various formulations of HS medium supplemented with the selected fruit processing wastewater fractions, as seen in Table 5. The thickness of the BC film samples, an important factor influencing their optical properties, was also measured.

The opacity of BC films varied according to the composition of the fermentation medium. The BC film produced in the HS commercial medium showed an opacity of 6.892 ± 0.042, which is consistent with previous studies indicating an opacity of 6.04 ± 0.50 for the HS control medium [14]. In turn, the BC films produced from HS medium supplemented with the selected fractions obtained by sequential membrane fractionation using raw wastewater revealed slight variation in opacity depending on the concentration and type of fraction used.

Samples supplemented with 5.0% (*v*/*v*) and 10.0% (*v*/*v*) of F#3 presented opacities of 7.862 ± 0.063 and 6.933 ± 0.116, respectively. The higher opacity related to the BC film produced from HS medium supplemented with 5.0% (*v*/*v*) of F#3 can be attributed to additional components in the retentate from the 25 kDa UF membrane, which increase light absorption. Notably, the higher concentration of F#3 (10% (*v*/*v*)) did not result in a proportional increase in opacity compared to 5.0% (*v*/*v*), and the opacity was not significantly different from the control HS medium, as seen in Table 5.

On the other hand, BC films produced from HS medium supplemented with 5.0% (*v*/*v*) and 10% (*v*/*v*) of F#6 exhibited significantly lower opacities of 3.049 ± 0.017 and 3.527 ± 0.094, respectively. The reduction in opacity can be attributed to removing larger particles from raw wastewater during NF, resulting in more transparent films. Additionally, the increase in transparency may be related to a more organized arrangement of cellulose chains, although F#6 exhibited only a slight increase in crystallinity compared to the HS control.

The WI of BC films also changed with the supplementation of the fermentation medium. The BC sample produced from the control HS medium showed a *WI* of 71.080 ± 3.622. BC films supplemented with 5.0% (*v*/*v*) and 10.0% (*v*/*v*) of F#3 in the HS medium exhibited higher *WI*s (75.382 ± 1.475 and 75.859 ± 1.552, respectively), indicating that the addition of F#3 enhanced the white appearance of the BC films, as seen in Table 5.

Similarly, BC films produced from HS medium supplemented with F#6 showed *WI*s of 75.506 ± 1.729 and 73.307 ± 2.501, respectively. Despite a slight, non-significant reduction in *WI* when 10.0% (*v*/*v*) of F#6 was added to the HS medium, both samples maintained or slightly improved their whiteness compared to the control sample.

On the other hand, the color change in the samples was measured in terms of Δ*E*, with the CB film produced from the HS medium acting as the control sample without color change data. Samples made from HS medium supplemented with F#3 and F#6 showed slight color changes, with the sample supplemented with 10.0% (*v*/*v*) of F#3 showing the highest change (Δ*E* = 5.630 ± 1.051) and the sample supplemented with 10.0% (*v*/*v*) of F#6 showing the lowest (Δ*E* = 3.132 ± 1.603), as seen in Table 5. These results suggest that the type and concentration of supplementation play a crucial role in determining the color stability of BC films.

Therefore, these findings are consistent with the literature, which highlights variations in opacity and whiteness depending on the composition of the fermentation medium and the presence of impurities or additives [14]. Thus, BC films produced from HS medium supplemented with F#3 are preferred for applications demanding high opacity, like food packaging. In contrast, those supplemented with F#6 are more suitable for transparent applications, such as electronics.

#### 3.3.2. Fourier Transform Infrared Spectroscopy (FTIR)

**Figure 3:**
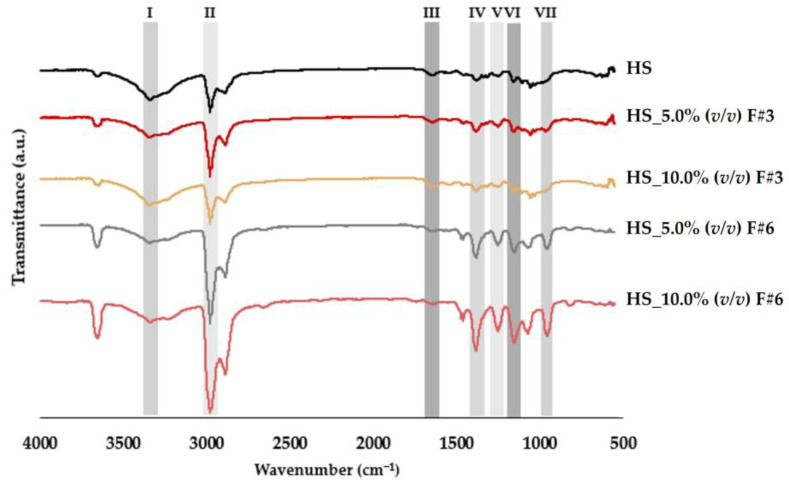
ATR–FTIR spectral analysis of BC films produced from HS medium supplemented with fruit processing wastewater fractions.

The chemical structure of BC films, produced using the HS medium supplemented with the selected fractions from raw fruit processing wastewater after sequential membrane fractionation, was analyzed by FTIR, as illustrated in Figure 3. All FTIR spectra displayed characteristic bands of BC, consistent with findings in the literature and showing considerable similarity across samples. A notable broad peak around 3330 cm^−1^ (I), associated with O–H stretching, indicated the presence of intra- and inter-chain hydrogen-bonded hydroxyl groups (–OH) [4,5,6,14,58]. Additionally, a strong absorption band near 2900 cm^−1^ (II) corresponded to C–H stretching vibrations typical of cellulose type I, involving CH_2_ and CH_3_ groups, while bands in the 1250 to 1400 cm^−1^ range (IV and V) suggested C–H and CH_2_ bending vibrations [4,5,6,14]. Bands observed around 1640 cm^−1^ (III) in the BC films were linked to the bending vibrations of absorbed water molecules (H–O–H) [5,6,14,58]. Finally, the band at 950 cm^−1^ (VII) corresponded to the stretching vibrations of the C–O–C bond in the β-1,4-glycosidic linkages, indicating antisymmetric out-of-phase ring stretching between glucose units in cellulose, while the band around 1150 cm^−1^ (VI) was linked to the antisymmetric bridge stretching of the C–O–H bond in 1,4-β-glucoside [4,5,6,14].

Therefore, these results suggest that BC produced from various feedstocks maintains a consistent chemical structure, which is crucial given the potential to substitute or supplement fermentation media with by-products from various industries. This finding could enhance the efficiency and viability of BC production, addressing the cost challenges associated with synthetic commercial HS media. Similar outcomes have also been reported in other studies who have explored alternative low-cost substrates, such as liquid tapioca waste where almost no difference was observed in the FTIR spectra compared to the standard HS medium [5]. Zhao et al. (2018) showed also that the FTIR spectrum of BC obtained in HS medium and BC in polysaccharide fermentation wastewater after 4 days and 10 days of fermentation exhibited a slight variation in peak intensity but shared a distinctive shape in common [23]. Furthermore, other researchers have successfully used acerola by-product and agricultural waste products, like pineapple peel extract, banana extract, and mixed pineapple peel and banana extracts, demonstrating promising results in maintaining similar BC characteristics as confirmed through FTIR analysis, while also reducing production costs [58,59].

These advancements could pave the way for broader adoption and commercialization of BC, which is currently limited to a few companies in the United States, Canada, Brazil, and Poland and focuses predominantly on biomedical applications [58].

#### 3.3.3. Transmission Electron Microscopy (TEM)

A detailed characterization of the nanostructural properties of BC films, produced using various HS media formulations supplemented with the selected fractions derived from fruit processing raw wastewater through sequential membrane fractionation, was examined via TEM.

**Figure 4:**
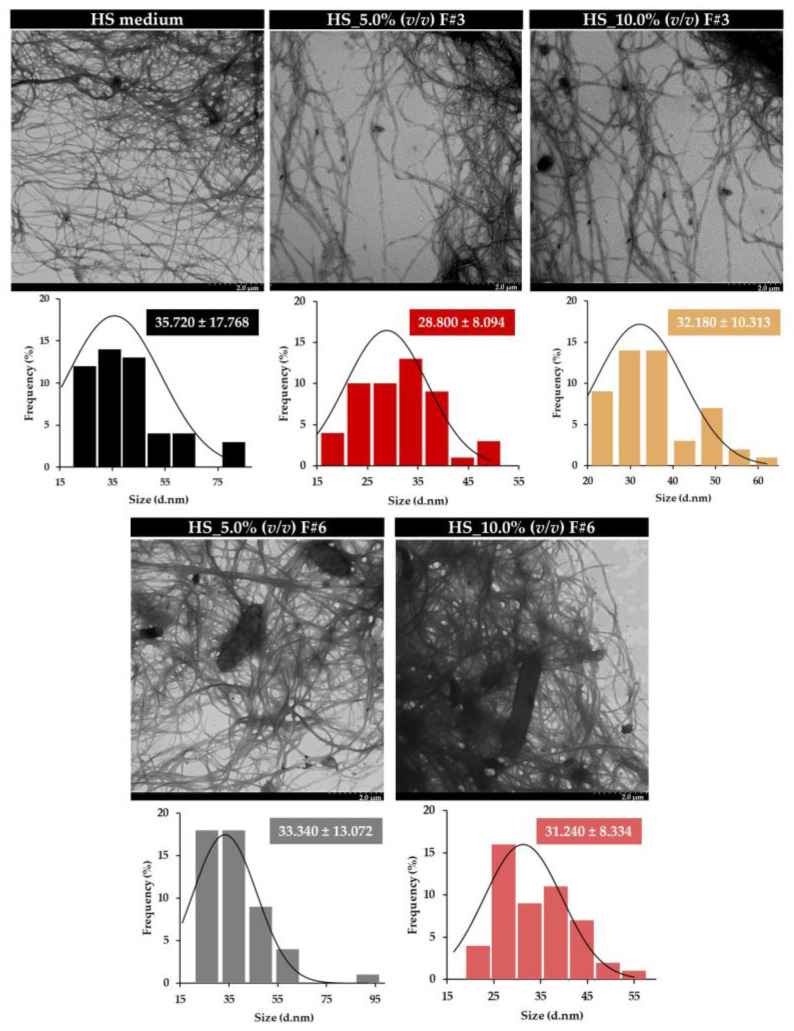
TEM images of BC films produced on HS media supplemented with fruit processing wastewater fractions.

The TEM images showed the presence of cellulose nanofibrils, which form dense, interwoven networks, typical of the BC structure. Moreover, as the production progressed over seven days, the microorganisms became encapsulated within the fibers they produced, continuing to secrete cellulose and resulting in increasingly compact BC structures, as shown in Figure 4 [4].

Moreover, the BC films produced on HS media with different processing wastewater fractions showed no significant changes in nanofibril diameters. As shown in Figure 4, the average nanofibril diameters ranged from 28.800 ± 8.094 nm to 35.720 ± 17.768 nm, which aligns with the typical range for natural BC nanofibrils (20–150 nm) [60]. This confirms that the supplementation did not significantly affect the nanoscale structure of the produced BC films.

#### 3.3.4. X-Ray Diffractometry (XRD)

**Figure 5:**
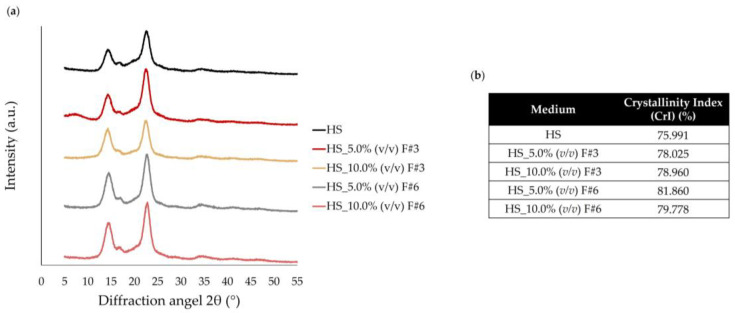
Characterization of the BC films produced on HS media supplemented fruit processing wastewater fractions via XRD analysis: (**a**) diffraction patterns and (**b**) crystallinity index (CrI) (%).

X-ray diffraction was used to analyze the crystalline structure and changes in the degree of crystallinity of the BC produced from different fermentation media. The X-ray patterns of the BC samples are displayed in Figure 5a. All samples exhibited three characteristic diffraction peaks of native BC at approximately 2θ = 14.5°, 16.8°, and 22.5°, corresponding to the (100), (010), and (110) crystallographic planes, respectively. As visible in Figure 5a, the intensity of the 100 reflections exceeds that of the 010 reflections when the film is oriented parallel to the X-ray beam, while this relationship is inverted in the perpendicular orientation. This indicates a pronounced uniplanar alignment resulting from the cellulose ribbons aligning parallel to the film surface during drying.

Moreover, the crystallinity index of the BC films produced by supplementing the HS medium with 5.0% (*v*/*v*) and 10.0% (*v*/*v*) of F#3 and F#6 ranged from 78.025% to 81.860%, which was higher than the crystallinity index of the HS control media. The lower crystallinity in the BC film from the HS control medium might be attributed to its slightly lower mechanical strength than the films from the supplemented medium (see further details in Section 3.3.6). This suggests that adding fractions from raw wastewater resulted in a slightly visible increase in crystallinity, as shown in Figure 5b.

The BC samples supplemented with the F#3 (HS_5.0% (*v*/*v*) F#3 and HS_10.0% (*v*/*v*) F#3) showed an increase in crystallinity to 78.025% and 78.960%, respectively. This indicates that the F#3 fraction contributed to a more crystalline BC structure, with the higher concentration (10.0% (*v*/*v*)) leading to a slightly higher crystallinity compared to 5.0% (*v*/*v*). In turn, the BC samples supplemented with the F#6 fraction (HS_5.0% (*v*/*v*) F#6 and HS_10.0% (*v*/*v*) F#6) showed even higher crystallinity degrees, with HS_5.0% (*v*/*v*) F#6 reaching 81.860% and HS_10.0% (*v*/*v*) F#6 at 79.778%, as seen in Figure 5b.

Therefore, the supplementation of the HS medium with various fractions from raw wastewater showed a slight increasing trend in the crystallinity of the produced BC films, with the F#6 at 5.0% (*v*/*v*) proving to be the most effective. Similarly, Zhang et al. (2024) observed a slight difference in BC crystallinity when comparing BC production using the HS medium and rice soaking wastewater (RSW) [61].

Moreover, this higher crystallinity, associated with improved mechanical strength and thermal stability, could be advantageous for specific BC applications. These findings highlight how fermentation medium composition influences BC properties [62]. Similar trends have been observed using alternative feedstocks, like apple waste [14] and bread waste [62], further supporting these results. Moreover, Revin et al. (2021) explored molasses as an alternative substrate and their results showed that BC samples exhibited a crystallinity of 83.02%, higher than that of standard HS medium (79.7%), which aligns with our findings (CrI_HS_: 75.991%; CrI_HS_5.0%(*v*/*v*) F#3_: 78.025%; CrI_HS_10.0%(*v*/*v*) F#3_: 78.960%; CrI_HS_5.0%(*v*/*v*) F#6_: 81.860%; CrI_HS_10.0%(*v*/*v*) F#6_: 79.778%) [63].

Such insights pave the way for the development of new and improved BC-based materials tailored to specific applications.

#### 3.3.5. Differential Scanning Calorimetry (DSC) Measurements

**Figure 6:**
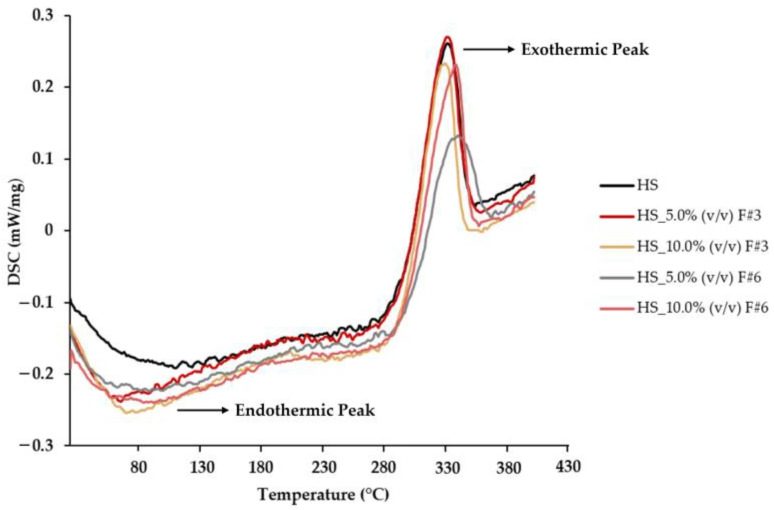
DSC patterns of the BC films produced using HS media supplemented with the fractions obtained from fruit processing wastewater through sequential membrane fractionation.

DSC analysis was employed to evaluate the thermal stability of BC films produced from HS media supplemented with the different fractions obtained from fruit processing raw wastewater from sequential membrane fractionation. Figure 6 illustrates the DSC curves, detailing the thermal behavior of the BC films by showing the heat absorbed or released as a function of temperature.

The DSC profiles of BC samples from different formulations displayed two distinct peaks with a similar pattern. The initial peak, occurring at approximately 80 °C, corresponds to the evaporation of residual water from the BC biopolymer, consistent with the previous literature [14,64]. The second peak, observed around 330 °C, indicates cellulose’s thermal degradation (pyrolysis), attributed to the breakdown of carbonyl and carboxyl groups [64].

Interestingly, for the BC films produced from HS media supplemented with the F#6, the exothermic peaks were observed at slightly higher temperatures than the other samples, indicating a potential increase in thermal stability. This suggests that while the overall thermal stability of the BC films remained relatively unchanged, the addition of F#6 could slightly enhance the thermal stability of the BC films. Therefore, these films could be more suitable for applications requiring higher thermal stability.

#### 3.3.6. Mechanical Properties

The properties of the BC are closely tied to the specific architecture of its cellulose synthase machinery, which involves both intracellular biosynthesis and extracellular assembly mechanisms [1]. This architecture profoundly influences the arrangement of microfibrils and the extent of hydrogen bonding between filaments, affecting the films’ mechanical performance. As a result, several studies indicate that BC films often exhibit brittle behavior, characterized by high tensile strength and Young’s modulus but with low elongation at break. Various factors, including the type of fermentation conditions and film processing methods, such as drying techniques, influence these mechanical properties [1]. Therefore, the values of tensile strength, elongation at break, and Young’s modulus of the dried BC films produced from the HS medium supplemented with the selected fractions obtained from fruit processing raw wastewater after sequential membrane fractionation were determined to evaluate their effects on the mechanical properties of the BC films.

The results are summarized in Table 6, which presents the comparative analysis of the mechanical properties for BC films produced under different conditions.

The data demonstrated that fractions obtained from fruit processing raw wastewater after sequential membrane fractionation significantly impact the mechanical properties of BC films. The BC sample produced from the HS medium supplemented with 5.0% (*v*/*v*) F#3 resulted in an increase in tensile strength (20.132 ± 5.656 MPa) and Young’s modulus (3706.835 ± 1420.495 MPa) compared to the sample produced with the control HS medium, as seen in Table 6. This increase can be attributed to the uniform and continuous network of cellulose fibrils interleaved by acetic acid bacteria, as mentioned in a previous study conducted by Tapias et al. [1].

Moreover, the BC film produced from HS supplemented with 5.0% (*v*/*v*) F#6 exhibited the highest increase in tensile strength (23.149 ± 4.041 MPa) and Young’s modulus (4827.031 ± 2171.323 MPa) among all formulations, indicating an enhancement in the film’s stiffness and strength, as seen in Table 6. This finding supports that the cellulose fibril network is more well-structured and continuous in this sample.

In addition, all films supplemented with fractions exhibited lower elongation at break values compared to the HS control. This suggests that while the fractions can enhance the stiffness and strength of BC films, they also tend to reduce their elasticity, making them more brittle and less capable of deforming before breaking. This could be related to a slight increase in crystallinity compared to the HS control, which leads to a more ordered and rigid molecular structure, restricting the mobility of cellulose chains. For example, the BC films supplemented with 5.0% (*v*/*v*) F#6 exhibited the highest crystallinity (81.860%) and the lowest elongation at break (0.545 ± 0.225%), indicating an inverse correlation between crystallinity and flexibility. Likewise, BC films supplemented with 10.0% (*v*/*v*) F#6 showed slightly lower crystallinity (79.778%) but an increase in elongation at break (1.182 ± 0.185%), reinforcing the trend that higher crystallinity reduces flexibility.

These findings align with the existing literature, providing a robust confirmation of the observed trends. BC films, as noted in the literature, generally have high tensile strength and Young’s modulus but limited elongation at break. For instance, Tapias et al. observed similar trends with oregano-derived films achieved a Young’s modulus of 925.6 ± 77.0 MPa and a maximum tensile strength of 30.3 ± 4.2 MPa [1]. Moreover, Zhao et al. (2018) reported that the strain at break increased from 3.40 ± 0.67% to 3.77 ± 0.90% for BC samples obtained in HS medium after 4 and 10 days of fermentation, respectively [23]. In contrast, for BC samples obtained in fermentation wastewater, lower strain at break values of 1.18 ± 0.39% and 1.85 ± 0.05% were observed after 4 and 10 days of fermentation, respectively [23]. Similarly, this study followed a higher elongation at break value for BC produced in HS medium (1.514 ± 0.181%) after 7 days of fermentation.

#### 3.3.7. Water Holding Capacity (WHC)

BC’s high water retention capacity, attributed to its hydrophilicity and high surface area-to-mass ratio, is essential for various applications, particularly in the biomedical field. In these applications, retaining moisture and absorbing exudate is critical for the effectiveness of wound dressings and tissue engineering materials. The WHC, which measures the water weight per unit weight of cellulose fibrils, is thus crucial for BC’s use in drug delivery and wound healing applications. In addition, BC’s high surface area, hydrophilicity, porous nature, and loose fibril arrangement enhance its WHC, making it also valuable for use in food packaging, paper production, immobilization agents, and various other industries [6,14].

**Figure 7:**
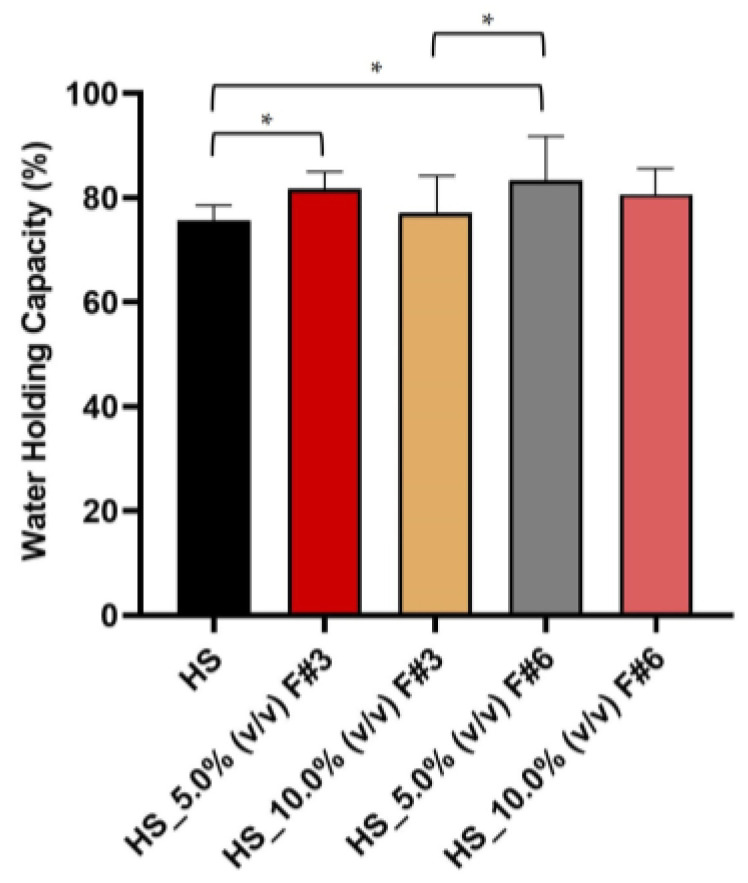
Water holding capacity (WHC) of the BC films produced on HS media supplemented with fruit processing wastewater fractions. (Data are presented as the mean ± standard deviation (SD), * *p* < 0.05).

Therefore, the WHC of the BC films produced from different HS medium formulations supplemented with various fractions was evaluated. It was found that the BC produced from the HS medium exhibited a WHC value of 75.548% ± 2.934%, as shown in Figure 7. In comparison, the BC films supplemented with 5.0% (*v*/*v*) F#3 showed a significant increase in WHC value to 81.707% ± 3.259%, while those supplemented with 10.0% (*v*/*v*) F#3 demonstrated a WHC value of 77.094% ± 7.025%, indicating an improvement but not as pronounced as observed with 5.0% (*v*/*v*) F#3. Similarly, the BC films supplemented with 5.0% (*v*/*v*) F#6 exhibited an improved capacity to retain water, with a WHC value of 83.306% ± 8.385%, highlighting their potential for enhanced performance in targeted applications. However, adding 10.0% (*v*/*v*) F#6 to HS medium resulted in a WHC value of 80.752% ± 5.091% which, while higher than the HS control, did not surpass the formulation with 5.0% (*v*/*v*) F#6.

Therefore, these results indicated that the addition of F#3 and F#6 fractions to the HS medium can enhance the WHC of BC films, with the 5.0% (*v*/*v*) F#6 showing the highest efficacy.

These findings align with previous studies, which report that BC exhibits a high WHC of 98.8% ± 0.3% [65], with other reports indicating 95.1% and 92.5% WHC values [6]. This is a crucial characteristic due to its porous, fibrillary, and homogeneous structure that creates capillary forces to trap water molecules. The extensive hydrogen bonding between cellulose fibrils and water molecules further enhances water retention [65]. However, although the WHC values obtained in the present research are slightly lower, they remain within a comparable range. This confirms that BC’s structural properties are essential for its water retention capabilities and support its effectiveness in various applications, particularly in biomedical and food packaging fields. Furthermore, variations in the fermentation media, drying processes, and the modifications or additives used during BC production may explain the observed differences in WHC values.

## 4. Conclusions

This research study explored fruit processing wastewater by kombucha SCOBY as an alternative substrate for BC production. The focus was on the impact of C and N content on BC yield and properties by supplementing the commercial HS medium with fractions from sequential membrane treatment of the raw wastewater.

The raw wastewater exhibited a high COD value of 12.85 ± 0.60 g/L, an alkaline pH of 11.5 ± 0.8, and a high concentration of suspended solids (2.52 ± 0.09 g/L), unfavorable for BC production. Conversely, sequential membrane fractionation resulted in F#1 with a high COD of 23.32 ± 1.64 g/L, inhibiting microbial activity and hindering BC production. In contrast, F#3, with a lower COD (15.26 ± 0.51 g/L), provided better conditions for BC production, while F#6, with a COD of 0.76 ± 0.03 g/L, which closely matches its reducing sugar content (0.74 ± 0.06 g/L), offers a more refined option for BC production.

Therefore, in evaluating these alternatives, it was found that supplementation with 5.0% (*v*/*v*) and 10.0% (*v*/*v*) of F#6 yielded remarkable results, demonstrating that the superior nutrient quality of F#6 can effectively enhance BC production. In addition, BC films supplemented with F#3 exhibited increased opacity, making them appropriate for applications requiring light barrier properties, such as food packaging, while BC films supplemented with F#6 displayed lower opacity, making them more suitable for transparent applications like electronics.

Furthermore, FTIR analysis confirmed that the BC samples maintained a consistent chemical structure across all formulations, indicating that alternative substrates like F#3 and F#6 do not compromise the chemical integrity of the BC. Complementing this, TEM analyses showed that BC films produced with F#3 and F#6 retained their characteristic nanofibril structures, underscoring the robustness of BC production across different formulations. In contrast, XRD analysis revealed that BC films supplemented with F#3 and F#6 exhibited a slight increasing trend in the crystallinity compared to the HS control. This enhanced crystallinity is linked to improved material properties, which are advantageous for applications in biomedical devices, packaging materials, and composites. However, further analysis would be valuable in evaluating the influence of any residual substances on the safety and efficacy of BC for specific applications. Additionally, DSC analysis demonstrated consistent thermal stability across all BC samples, with a slight increase observed for F#6, indicating that the presence of wastewater fractions can enhance the thermal properties of the BC films.

Mechanical testing further elucidated the performance of the produced BC films, revealing that those supplemented with F#3 and F#6 demonstrated higher tensile strength and Young’s modulus. These results indicate enhanced mechanical stiffness and stability, making the F#3 and F#6-supplemented BC films particularly suitable for demanding applications requiring superior material performance. Moreover, WHC analysis revealed that both F#3 and F#6 significantly improve the WHC of BC films. Namely, BC films with 5.0% (*v*/*v*) F#3 and F#6 showed the highest water retention. These results demonstrated that adding the wastewater fractions enhanced the water retention capabilities of the BC films, which is beneficial for applications requiring high moisture retention.

Therefore, fruit processing wastewater, particularly fractions obtained through sequential membrane fractionation, presents a viable alternative to supplement the commercial HS medium for BC production. This approach enhances the sustainability of BC production by using industrial waste, effectively reducing costs associated with commercial synthetic media, particularly when using F#6. In this context, membrane fractionation transforms a typically expensive waste disposal process into a valuable resource recovery opportunity, aligning with the principles of the circular economy. Thus, membrane treatment of wastewater generated by fruit processing not only decontaminates the effluents, but also makes them suitable for BC supplementation, further reducing their environmental impact. However, although these fractions contain lower amounts of reducing sugars than the glucose content in the commercial HS medium, their sugar concentration, particularly in F#6, can be modified or enriched with sugars from other waste sources, such as agro-industrial by-products, to enhance their effectiveness and improve BC yield, potentially allowing the complete substitution of the commercial medium. Thus, future research should focus on optimizing nutrient composition and further exploring this alternative substrate’s scalability and economic feasibility in industrial BC production.

The microbiological indicators, including total coliforms, fecal coliforms, *Escherichia coli*, and fecal *Streptococci*, would be particularly relevant in future studies to ensure microbial safety when employing fruit processing wastewaters in industrial processes. Additionally, evaluating the stability of wastewater fractions during storage is crucial to ensure their practical use in BC production. Moreover, studying how seasonal variations in wastewater composition affect BC yield could enable targeted adjustments for improved outcomes.

Additionally, exploring BC’s unique properties produced from these alternative substrates could reveal new applications, leveraging their distinctive characteristics for specialized uses in various industries. Furthermore, polyphenols in fruit processing wastewater, concentrated through sequential membrane fractionation, can neutralize free radicals and reduce oxidative stress, offering beneficial antioxidant properties. This potential can enhance the BC produced from this wastewater, adding value and functionality to the materials.

## Figures and Tables

**Table 1:** Characteristics of the membrane modules used (spiral wound modules with 0.55 m^2^ of membrane area).

Process	Designation	Selectivity ^(1)^	Hydraulic Permeability (L/m^2^/h)	Operation Limits
MF	MFP5	0.5 µm	264	pH: 1–11 Pressure: 1–3 bar Temperature: 5–60 °C
UF	GR40PP	100 kDa	91	pH: 2–12 Pressure: 1–10 bar Temperature: 5–75 °C
	GR60PP	25 kDa	36	
	GR90PP	5 kDa	29	
NF	NFT	>99%	7	pH: 3–9 Pressure: 15–35 bar Temperature: 5–50 °C

^(1)^ Membrane selectivity according to the pore size for MF membranes, molecular weight cut-off (MWCO) for the UF membranes, and slat rejection with an aqueous solution of 2 g/L of MgSO_4_ for the NF membrane.

**Table 2:** Composition of each medium supplemented with fractions obtained from fruit processing raw wastewater.

Media Designation	Carbon and Nitrogen Sources
HS	2.0% (*w*/*v*) Glucose
HS_5.0% *(v*/*v)* F#1	2.0% (*w*/*v*) Glucose 0.5% (*w*/*v*) Peptone 0.5% (*w*/*v*) Yeast extract 5.0% (*v*/*v*) F#1
HS_10.0% *(v*/*v)* F#1	2.0% (*w*/*v*) Glucose 0.5% (*w*/*v*) Peptone 0.5% (*w*/*v*) Yeast extract 10.0% (*v*/*v*) F#1
HS_5.0% *(v*/*v)* F#3	2.0% (*w*/*v*) Glucose 0.5% (*w*/*v*) Peptone 0.5% (*w*/*v*) Yeast extract 5.0% (*v*/*v*) F#3
HS_10.0% *(v*/*v)* F#3	2.0% (*w*/*v*) Glucose 0.5% (*w*/*v*) Peptone 0.5% (*w*/*v*) Yeast extract 10.0% (*v*/*v*) F#3
HS_5.0% *(v*/*v)* F#6	2.0% (*w*/*v*) Glucose 0.5% (*w*/*v*) Peptone 0.5% (*w*/*v*) Yeast extract 5.0% (*v*/*v*) F#6
HS_10.0% *(v*/*v)* F#6	2.0% (*w*/*v*) Glucose 0.5% (*w*/*v*) Peptone 0.5% (*w*/*v*) Yeast extract 10.0% (*v*/*v*) F#6

**Table 3:** Characteristic parameters of the raw fruit processing wastewater.

Parameter (Units)	(Mean ± Standard Deviation)
pH	11.5 ± 0.8
Electrical conductivity (mS/cm)	1.80 ± 0.07
Total suspended solids (g/L)	2.52 ± 0.09
Chemical oxygen demand (COD) (g/L)	12.85 ± 0.60
Total dissolved nitrogen (N) (mg/L)	6.44 ± 0.31

**Table 4:** Characteristic parameters of the selected fractions after sequential membrane fractionation of the fruit processing wastewater. (Data are presented as the mean ± standard deviation).

Parameter (Units)	F#1 (>0.50 µm)	F#3 (5–25 kDa)	F#6 (<0.50 kDa)
pH	6.08 ± 0.27	5.98 ± 0.16	6.70 ± 0.23
Chemical oxygen demand (g/L)	23.32 ± 1.64	15.26 ± 0.51	0.76 ± 0.03
Reducing sugars (g/L)	0.78 ± 0.09	0.85 ± 0.11	0.74 ± 0.06
Total dissolved nitrogen (mg/L)	n.d.	6.18 ± 0.15	2.70 ± 0.07

n.d.: Not defined.

**Table 5:** Visual characteristics of the bacterial cellulose (BC) films produced from Hestrin–Schramm (HS) medium supplemented with various fractions from fruit processing raw wastewater after membrane fractionation: thickness, opacity, whiteness, and color change.

Samples	Thickness (mm)	Opacity (Abs 600 nm mm^−1^)	Whiteness Index (*WI*)	Color Change (Δ*E*)
HS	0.424 ± 0.050	6.892 ± 0.042	71.080 ± 3.622	-
HS_5.0% (*v*/*v*) F#3	0.326 ± 0.040	7.862 ± 0.063	75.382 ± 1.475	4.705 ± 1.528
HS_10.0% (*v*/*v*) F#3	0.439 ± 0.195	6.933 ± 0.116	75.859 ± 1.552	5.630 ± 1.051
HS_5.0% (*v*/*v*) F#6	0.871 ± 0.234	3.049 ± 0.017	75.506 ± 1.729	4.505 ± 1.502
HS_10.0% (*v*/*v*) F#6	0.908 ± 0.181	3.527 ± 0.094	73.307 ± 2.501	3.132 ± 1.603

**Table 6:** Characterization of the mechanical properties of the BC films produced on HS media supplemented with fruit processing wastewater fractions.

	Tensile Strength (MPa)	Young’s Modulus (MPa)	Elongation at Break (%)
HS	12.771 ± 4.445	837.633 ± 269.340	1.514 ± 0.181
HS_5.0% (*v*/*v*) F#3	20.132 ± 5.656	3706.835 ± 1420.495	0.647 ± 0.413
HS_10.0% (*v*/*v*) F#3	19.221 ± 7.099	1986.560 ± 1019.253	1.099 ± 0.605
HS_5.0% (*v*/*v*) F#6	23.149 ± 4.041	4827.031 ± 2171.323	0.545 ± 0.225
HS_10.0% (*v*/*v*) F#6	22.755 ± 8.449	1645.328 ± 870.893	1.182 ± 0.185

## Data Availability

Data that support the findings of this study are included in the article.
